# Supplementary material for: Evolutionary emergence of Hairless as a novel component of the Notch signaling pathway
Source: eLife. 2019 Sep 23;8:e48115. doi: 10.7554/eLife.48115 (PMC6777938; doi:10.7554/eLife.48115)
Supplement: Supplementary file 3. — Colors denote conserved sequence features. Motifs for direct recruitment of the CtBP and Gro co-repressor proteins (aligned in Table 1) are shown in red and green, respectively. Large region highlighted in orange is the highly conserved body of Su(H), extending from ‘LTREAM’ to ‘YTPEP’. [file elife-48115-supp3.docx]

**Supplementary file 3.** **Full-length Su(H) sequences**

>Ecdyonurus insignis (mayfly; Arthropoda; Ephemeroptera)

MQEDGGSGQAMTDLSAAGPGDQQQQQQQQQQQQQQQQQTSAGASGGGGGGGSTSHLTNGHAQSGPGPYPDNHPVDLSSPRPHDALDNGTTYHRDRRSADQYKQNGVDSEWQSPEVKSYGYPRWTVPWPPHASHSPRGTYWSDSVEPPNSSDPGAPMIPGSLTPPDKMNGEHPHHGAPGAPHPGAAAAAAAAAAMSHHFGLGAASMAAHGLQTPPSPIPTPSPPVPIERFSSLLYRPKDQRLTREAMKKYLRERSDMVIVILHAKVAQKSYGNEKRFFCPPPCIYLYGEGWRMKQEQMLKDGESEQGAQLCAFIGIGNSDQDMQQLDLNGKQYCAAKTLYISDSDKRKHFMLSVKMFYGNGHDIGVFHSKRIKVISKPSKKKQSLKNADLCIASGTKVALFNRLRSQTVSTRYLHVENGNFHASSTQWGAFTIHLLDDNESESEEFTVRDGYIHYGSTVKLVCSVTGMALPRLIIRKVDKQMALLDADDPVSQLHKCAFYMKDTERMYLCLSQERIIQFQATPCPKEPNKEMINDGASWTIISTDKAEYQFFEGMGPVRAPVTPVPVVSTLHLNGGGDVAMLELTGENFTPTLKVWFGDVEAETMYRCQESMLCVVPEISAFRGEWLWVRQPTQVPVSLVRNDGIIYATGLTFTYTPEPGPRPHCPAADDIMRNVTSHVNRVSGGPLGISYEQSASAQGGM

>Calopteryx splendens (damselfly; Arthropoda; Odonata)

MCGKRSPNMAMPSISPRGWNCRVSGCLGGASSPFRSLPQDDGDQGAVISDSAGGAHCEEEAGGGAVEEVEEGRRLEQLEVGGGRGPPGSGPYTDNHPVDLSSPRPPDAVAGDGAAYQRPRGLPENNYKANGLLADAEWQTQEAKHYGYPRWAVPWPLHSSHSPRTASYWPDMAASDASLAAADPSLHHMIPGSLTPPDKMNGEHPAMHQHHVPPPPAPPGSLHAPHHVALAAGASGGRGPGGAVSGPLLGPPSMAAHSMQTPPSPIPTPSPPVPMERFGSVLYRPKEQRLTREAMKKYLRERNDMVIVILHAKVAQKSYGNEKRFFCPPPCIYLYGEGWRLKQEQMLKDGESEQGAQLCAFIGIGNSDQDMQQLDLNGKQYCAAKTLYISDSDKRKHFMLSVKMFYGSGHDIGVFHSKRIKVISKPSKKKQSLKNADLCIASGTKVALFNRLRSQTVSTRYLHVENGNFHASSTQWGAFTIHLLDDNESESEEFTVRDGYIHYGSTVKLVCSVTGMALPRLIIRKVDKQMALLDADDPVSQLHKCAFYMKDTERMYLCLSQERIIQFQATPCPKEPNKEMINDGASWTIISTDKAEYQFFEGMGPVRSSVTPVPIVHSLHLNGGGDVAMLELTGENFTPNLKVWFGDVEAETMYRCQESMLCVVPEISAFRGEWLWVRQPTQVPVSLVRNDGIIYATGLTFTYTPEPGPRPHCPSADEIMRSGQTHLSRLPAITDVPGIQYDQQPSSQGGM

>Atelura formicaria (silverfish; Arthropoda; Zygentoma)

MTDLSTAGPGPGAPGSQQQQGSLHLNGHVGPYPDNHPVDLSSPRPQDALDAGYRDRRLPDQYKQNGLVGVPEPEWQTPEAKPYGYPRYPGPHMIPGSLTPPDKMNGEHPHHSHSHGPHPGSAAMSHHFGLPSMAHSMQTPPSPIPTPSPPVPIERFSSLLYRPKDQRLTREAMKKYLRERSDMVIVILHAKVAQKSYGNEKRFFCPPPCIYLFGEGWRLKQEQMLKDGETEQGAQLCAFIGIGNSDQDMQQLDLNGKQYCAAKTLYISDSDKRKHFMLSVKMFYGNGHDIGVFHSKRIKVISKPSKKKQSLKNADLCIASGTKVALFNRLRSQTVSTRYLHVENGNFHASSTQWGAFTIHLLDDNESESEEFTVRDGYIHYGSTVKLVCSVTGMALPRLIIRKVDKQMALLDADDPVSQLHKCAFYMKDTERMYLCLSQERIIQFQATPCPKEPNKEMINDGASWTIISTDKAEYQFFEGMGPVRSPVTPVPVVHSLHLNGGGDVAMLELTGENFTPNLKVWFGDVEAETMYRCQESMLCVVPDISAFRGEWLWVRQPTQVPVSLVRNDGIIYATGLTFTYTPEPGPRPHCPSADEIMRSGQTHLSRMPAITDVAGVQYDQQPPPQGGM

>Machilis hrabei (bristletail; Arthropoda; Archaeognatha)

MTDLSTAGPGPGSQQQGTLHLNGHVGAGPGGPYPDNHPVDLSSPRPHDNLDVAYRERRDQYKHNGLGVVGIPEPEWQTPEAKPYGYSRYPGPHMLPGSLTPPDKMNGEHPHHGHGHSHSVHSGGTAMAHHFGLATGAALGISSMAHSMQTPPSPIPTPSPPVPVERYSSSLYRPKDQRLTREAMKKYLRERGDMVIMILHAKVAQKSYGNEKRFFCPPPCIYLYGEGWRRKQEQMLKDGETEQGAQLCAFIGIGNSDQDMQQLDLNGKQYCAAKTLYISDSDKRKHFMLSVKMFYGNGHDIGVFHSKRIKVISKPSKKKQSLKNADLCIASGTKVALFNRLRSQTVSTRYLHVENGNFHASSTQWGAFTIHLLDDNESESEEFTVRDGYIHYGSTIKLVCSVTGMALPRLVIRKVDKQMALLDADDPVSQLHKCAFYMKDTERMYLCLSQERIIQFQATPCPKEPNKEMINDGASWTIISTDKAEYQFFEGMGPVRLPVTPVPVVHSLHLNGGGDVAMLELTGENFTPNLKVWFGDVEAETMYRCQESMLCVVPDISAFRGEWLWVRQPTQVPVSLVRNDGIIYATGLTFTYTPEPGPRPHCPSADDIMRTGQAHLNRMPPIADVTGVAYDQAPQPQGPL

>Catajapyx aquilonaris (forcepstail; Arthropoda; Diplura)

MTELSPVGPGPGGQQQQQATTQQQQQQQPPPPQQQHGLNGHVYGESPTPTSTANNPVDLSSPRGSELEEAYQRAERREHAYRHHNGLSVGVGGTLQDLDPHSAPMIPGSLTPPDKVNGEHHSHHPHHTHSHPGAAPPLPHHFALPPSGLGAMGPPMQTPPSPIPTPSPPVPIDRFGTSLYRTKEQRLTRDAMKRYLRERGDLVLVILHAKVAQKSYGNEKRFFCPPPCIYLFGDGWRRKREQLLKEGEAEQGAQLCAFIGIGNSDQDMQQLDLNGKQYCAAKTLYISDSDKRKHFMLSVKMFYGNGHDIGVFHSKRIKVISKPSKKKQSLKNADLCIASGTKVALFNRLRSQTVSTRYLHVENGNFHASSTQWGAFTIHLLDDNESESEEFTVRDGYIHYGATVKLVCSVTGMALPRLIIRKVDKQMALLDADDPVSQLHKCAFYMKDTERMYLCLSQERIIQFQATPCPKEPNKEMINDGASWTIISTDKAEYQFYEGMGPVRSPVTPVPVVHSLHLNGGGDVAMLELTGENFSPSLKVWFGDVEAETMFRCQESMLCVVPDISAFRGEWLWVRQPTQVPVSLVRNDGIIYATGLTFTYTPEPGPRPHCPSVDDIIRTGGVGSGVAGPVGHLSRMQSLADASAVVQYEQPHHPPQQGAM

>Holacanthella duospinosa (springtail; Arthropoda; Collembola)

MPQIMDPTDRVTSPWNQFHVGLGNSYVGSEQTGSNNGTPADSWDVSPLQDEYKFTPFNNNTTTDSPSTPASVNQYYLQQNEICVESSDQQQSLSELSSNGQQQEQQELNLNGHIDYGQSEVGHGSGLIPVPVVSGSIVVPNSNPVDLSNPSPSRHPVLDERTQQRLHHNIHQTQHDHYKHLQPLGAILHNLDQPHTSNFVPGSLSPPERMNGNDPSLLHTHPHHLSHMSGSPALSTHFGLPHAGLMPHPVHTPPSPIPTPSPPVPFDRFSSSLYRGKEQRLTREAMKRYLRDRGDMILVIQHAKVAQKSYGNEKRFFCPPPCIYLYNDGWRRKRDQLLKEGETEQGAQLCAFIGIGSSEQDMQQLDISGKQYCAAKTLFISDSDKRKHFMLSVKMFYGNGQNIGVFQSKRIKVISKPSKKKQSLKNADLCIASGTKVALFNRLRSQTVSTRYLHVENGNFHASSTQWGAFTIHLVDDNESESEEFTVRDGYIHYGSTVKLVCSVTGMALPRLIIRKVDKQLALLDADDPVSQLHKCAFFMKDTDRMYLCLSQERIIQFQATSCPKEPNKEMINDGASWTIISTDKAEYQYFEGMGPVRSPVTPVPVVHNLNVNGGGDVAMLELTGESFSPNLRVWFGDVETETMFRCQESMLCVVPNISAFRDRSDWLWVRQPTQVPVSLVRSDGIIYGTGLTFTYTPEPGPHRHPCQPPDESMLRNSLAPPTLPHHHHIPLPVPQYGPIMSSSSSGRQPPSHMPMLHDGSSSTGQLHYDLSQAAAL

>Hyalella azteca (crustacean; Arthropoda; Amphipoda)

MTGLSGLPALSTPVSSYQDLPHQQLHHSNTPNPPPHHQHLDPQHHSAPNSQHNPPSSHHQSLGHRPVDLSQAPSPRPTHQPHHPHYHHLTAPAPQLQHHLHQQQSHTLPKSEPGCAAAMLAGSLTPPDKLNSDPQQQQQQQQQHPALTQHQTIPQHPQHPAQHHPSVSGPPSLPHHHPHLLPPVMPALHTPPSPLPTPSPPHYERYTTMKEQRLTREAMQRYLNERGDQTLVILHAKVAQKSYGNEKRFFCPPPCIYLFGDGWQRKRHEIARTASSEHDAQLCAFIGIGNSDQDMQQLDLNGKHYCAAKTLFISDSDKRKHFLLSVKMFWGSGTDIGVFHSRRIKVISKPSKKKQSLKNAELCIASGTKVALFNRLRSQTVSTRYLHVENGNFHASSTQWGAFTIHLLDENESESEEFTVKDGYIHYGATVKLVCSVTSMALPRLVIRKVDKQMALLDADDPVSQLHKCAFYMKDTERMYLCLSQERIIQFQATPCPKESNREMINDGASWTIISTDKAEYLFYEGMGPVRAPITPVPVVNTLHLNGGGDVAMLELTGESFTPMLRVWFGDVEAETMYRCQESMLCVVPDISAFRRGWQWVRQPTLVPITLVRNDGIIYATNLTFTYTPEPGPRQHCPQADAIMRPHRAPLAAAQQHGDLPGLQSSPPANPLVPLQFEPSQPLPPQALSQQHGGAPHNMNIMSHIQQQQQQQPQMQQHQQHPQSSQQQATHQQPQ

>Eurytemora affinis (crustacean; Arthropoda; Calanoida)

MLLNMEALVELPEIKSEIKAEDFAAQQQQQQHSVSVNSQQNCQWNQFGYGGEELWSSVYPGTPNSQHLVPGTPNSHLGSVTPNSHLGSVTPTSHLGSVTPTSHHAPPCTTPQTHHTLTNVITNNNAYFSQDPGLLAGYSETSAPVDLSAPRPNIHTDRYSAVDDWSGDKYNYGRYGMLPGSLTPPDKLNGDHCSPGLGPLPGSHWGGNLGLSSMAGPMQTPPSSPPMAIDRFGSSLYRSKEQRLTRDAMKKYLRERGDMVVVMLHAKVAQKSYGNEKRFFCPPPCIYLYGDGWRRKKEEMQRSGESEQGSQLCAFIGIGNSDQDMQQLDLNGKHYCAAKTLFISDSDKRKHFLLSLKMFYGNGTDIGVFHSKRIKVISKPSKKKQSLKNADLCIASGTKVALFNRLRSQTVSTRYLHVENGNFHASSVQWGAFTIHLLDDNESESEEFTVRDGYIHYGSSVKLVCSITGMALPRLIIRKVDKQMAHLDADDPVSQLHKCAFYMKDTDRMYLCLSQERIIQFQATPCPKEPNKEMLNDGANWTIISTDKVEYQFYEGMGPVRNPVTPVPVVHSLHLNGGGDVAMLELTGECFTPNLRVWFGDVESETMYRCQESMLCVVPDISQFRGGWQWVRQPTQVPVSLVRNDGIIYATGLTFTYTPEPGPRSHCTSADDIMRQGQNVDYNPASQVLM

>Triops cancriformis (shrimp; Arthropoda; Notostraca)

MPDAVPMDHLPPSARSPHDSWGQYGYGLSGYEAQQSRPDSDAGNPHSHSSTSNNEVELWNSSHSSQHHNPSSDPLSPYQGGTPLSPAGTPQQHHAYYSQNPGVVPSMTELGGSTNASLHSLTPHYLPALNNHPGGNPPSFHPEARPVDLSSSRLLAAGHAPHVLQQLGNPGTPLYLDTYNRDNRRDAYKQNGLSLSLGIGESEWQSPDPKTQNYLRYHSSSLTLTPPDKVNVDGSNSQSGGGGSQQQQLHSASSSYSIAVANMVPTLQTPPSPLSTPSPPVPLERYGGPPLFRPKEQRLTREAMMRYLQERGDMVLVILHAKVAQKSYGNEKRFFCPPPCIYLYGDGWRRRREQLQREATAAGASAAEAEASSQLCAFIGIGNSDQDMQQLDLNGKHYCAAKTLFISDSDKRKHFMLSVKMFYGNGQDIGVFHSKRIKVISKPSKKKQSLKNADLCIASGTRVALFNRLRSQTVSTRYLHVENNNFHASSTQWGAFTIHLLDDSESESEEFTVRDGYIHYGSTVKLVCSVTGMALPRLIIRKVDKQMAVLDADDPVSQLHKCAFYMKDTERMYLCLSQERIIQFQATPCPKEPNKEMINDGAAWTIISTDKAEYQFFEGMGPVRSPVTPVPIVHSLHLNGGGEVAMLELTGENLTPNLKVWFGDVEAETMFRCEESMLCVVPDISAFRSGWQWVRHPTQVPVSLVRSDGVIYATRLTFTYTPEPGPRPHCPTTDEVLRGSVSTNGSSHMVGGNMHYEHLPQGPTM

>Argulus siamensis (louse; Arthropoda; Arguloida)

MFDSNQGQYSYSLNSGYDNRGTPPEGVWHNVNGGESSYYAANSSESVPTTTGYYIKEENDDVSSNMTELGSTPAPHPPPQVQPSMHHHMNGHAPGGRYPENNPVDLSNSRTGLPELVYRDRRDIGQASHYKPNGLGLNVGIPTDSDWQNPEGKNHYGYPRYPAASPMIPGSLTPPDKMNGEHHPGHPNHHHPAPHHMMGIPTSSPLSISTMVSSMQTPPSPLPTPSPPVPLERFGLNCKDQRLTREAMKKYLRERGDMTIMILHAKVAQKSYGNEKRFFCPPPCIYLFGEGWKRKREQMLREGENEQGAQLCAFIGIGSSDQDMQQLDLSGKNYCAAKTLYISDSDKRKHFFLSVKMFYGNGHDIGVFESKRIKVISKPSKKKQSLKNADLCIASGTKVALFNRLRSQTVSTRYLHVENGNFHASSTQWGAFTIHLLDENESESEEFTVRDGYIHYGSTVKLVCSVTGMALPRLIIRKVDKQMAQLDADDPVSQLHKCSFYMKDSERMYLCLSQERIIQFQATPCPKEPNKEMLNDGAAWTIISTDKAEYQFFEGMGPVRAPVTPVPTVQSLHLNGGGDVAMLELKGENFTPNLRVWFGDVEADTMFRCQEGLLCVVPDISAFRGGWQWVRQPTQVPVSLVRHDGIIYATGLTFTYTPEPGPRNHCPQSLDVLRPGSISRNNIGQPDPAMGYDQIPPSHHPL

>Strigamia maritima (centipede; Arthropoda; Geophilomorpha)

MNEVFLPQGQYDYPPPLASTYSREADLWNVNLATYSSAPTTCTGATPAPSVTGFYAQATGSNSVSPSSVSLTTLTPHFADNHPVDLSNSHRGEGGHLDLVRFQSDRVDAYKHANGLSVHIPDHHDATSHMIAGSLTPPDKVNGEHGHQLVTMSNASQMSLGSIASSLQTPPSPIPTPSPPVPLDGVRHSHKDQRLTREAMKRYLRERGDQVLVILHAKVAQKSYGNEKRFFCPPPCIYLFGDGWRRKREQMLHEGETEQGAQLCAFIGIGNSDQDMQQLDLNGKNYCAAKTLYISDSDKRKHFMLTVKMFYGNGEDIGVFHSKRIKVISKPSKKKQSLKNADLCIASGTKVALFNRLRSQTVSTRYLHVENGTFHASSTQWGAFTIHLLDDNESESEEFTVRDGYIHYGSTVKLVCSVTGMALPRLIIRKVDKQTALLDADDPVSQLHKCAFYMKDTERMYLCLSQERIIQFQATPCPKEPNKEMINDGASWTIISTDKAEYTFCEGMGPVRTLVTPVPVVHSLHLNGGGDVAMLELTGENFTPTLRVWFGDVEAETMYRCQESMLCVVPDISAFRGGWQFVRQPTQVPVSLVRSDGIIYATGLTFTYTPEPGPRPHCPATDHILRGGSQSGLDRLPTSPDPALTYNPPHHALTPI

>Sigmoria latior munda (millipede; Arthropoda; Polydesmida)

MQGTASPATPVMSELSHTPIPGNNSHVHLNGNVQPRFTNENHPVDLSSSHRSSESPLHHRDRPPPVYKHPNGLNITIPDHQAEAPWHAPPYSGNNTELADASHMIPGSLTPPDKGNAEHSHSHHPHHHHPHHHHHHLVGISGSNPMAPPLQTPPSPLPTPSPPVPMDNVRHGYPWKPLHYSEVHSQQMHHLSHSEQRLTREAMRSYLRERGDQVLVILHAKVAQKSYGNEKRFFCPPPCIYLFGEGWRRKREQMLRDGENEQGAQLCAFIGIGNSEQDMQQLDLNGKAYCAAKTLYISDSDKRKHFMLTVKMFYGNGADIGVFYSKRIKVISKPSKKKQSLKNADLCIASGTKVALFNRLRSQTVSTRYLHVENGNFHASSSQWGAFTIHLLDDQESESEEFTVRDGYIHYGSTIKLVCSVSGMALPRLVIRKVDKQTALLDADDPVSQLHKCAFYMKDTERMYLCLSQERIIQFQATPCPKEPNKEMINDGASWTIISTDKAEYTFYEGMGPVRTPVTPVPVVQSLHLNGGGDVAMLELTGENFTPNLRVWFGEVEAETMYRCQESMLCVVPDISAFRGGWQWVRQPTQVPVSLVRSDGVIYTTGLTFTYTPEPGLRSHCSSAEQILRTTQPSIERYPHPADNSMINYGGHPQM

>Metaseiulus occidentalis (mite; Arthropoda; Mesostigmata)

MNDIYNSHHHLGEEAPYPTPYPTPYPTPYPQHAPVSIQNNNYDSSAFSASTSMAGSSLNPSPLYSDPRDHHLAMLDSYGADRKPLDMSAAHRSHPYFSAAHSPMHYRGHHPPPAHPEEPPMMIASLAGSNKDLNHKQHGLLGPPKLTGGHLPPSPLPSPPVNALARRKDQPLTRDVMARYLIERNSNDMVLVILHAKVAQKSYGNEKRFFCPPPCIYLFGDGWQRKQDQIMREYGDNTEAGHQASQLCAFIGIGNSDQDMQPLDFNGKKYCAAKTLFISDSDKRKHFMLSVKMFYGNGEDLGVFHSKRIKVISKPSKKKQSLKNADLCIASGTKVALFNRLRSQTVSTRYLHVENDNFHASSTQWGAFEIHLLDDEESESEEFNVRDGYIHYGSTVKLVCSVTGMAMPRLVIRKVDKQTALLDADDPVSQLHKCAFYMKDSERMYLCLSQEKIIPFQATQCPKEPNKEMINDGAAWTIISTDKAEYTFFEGMGPVRAPVTPVPVVNSLHLNGGGEIAMLELVGENLTPDLKVWFGNVEAPETMFRCEESMLCVVPDISAFKEGWQWVRQPTQVPVSLVRSDGVIYATSLTFVYTPEVGPRQPYPCVGDILRPQSQLQSVSPGCGQQQQASQLQDDVNSQQSQSSTNHYMHLQ

>Ixodes scapularis (tick; Arthropoda; Ixodida)

MSDVYLPDDQYAGYHPVQNNYSSPHDDGTFAVSGSAYSGDYGRDLLLDLGQQAAGAPVDMSSHPARAHPYFNSGGVPFKNGLADGEPGALLGSAAKGAGEGPPQGSPHLGVQAPRLPPSPLPSPPSEERYRRGEPRLTRDAMDRYLRDRGDMVLVILHAKVAQKSYGNEKRFFCPPPCVYLLGDGWQRKRDQLLRDGEADQAAQLCAFIGIGNSDQDMQQLDFAGKSYCAAKTLFISDSDKRKHFMLSVKLFYGNGEDVGVFQSKRIKVISKPSKKKQSLKNADLCIASGTRVALFNRLRSQTVSTRYLHVDGGNFHASSSQWGAFTIHLLDDNESEAEEFTVRDGYIHYGSTVKLVCSVTGMALPRLVIRKVDKQNAFLDADDPVSQLHKCAFYMKDTERMYLCLSQEKIIQFQATPCPKEPNREMINDGASWTIISTDKAEYTFHEGAGPVRVPVTPVPVVNSLHLNGGGDIAMLELTGENFAPNLRVWFGNVEAETMYRCAECLLCVVPDISAFREGWQWVRQPTQVPVSLVRSDGVIYATGLTFTYTPEPGPRQPGPYPALVHDILRPANARSHPPAPEDHGAPTANFGHHMHFGSHHQNMS

>Parasteatoda tepidariorum 1 (spider; Arthropoda; Araneae)

MSLAPYTTAYSSASSTPSQNASSYSITSQANLQNPNPYTDLNVNNRAQHQQLGHVQLNEVNRNGMQNSHLCSPSRDRPSENVIDSHPVDLSSPKPSNRYGPMGYMNVARDNSTGAFPNFNNRSGFENGMDSMRPEYNQKLNGEQNQQQPHHPLVPHGALSLPLLSQGRGHAPPSPMPTPSPPVDDRHRSKRKDQRLTRECMKKYLRERGDMVLVILHAKVAQKSYGNEKRFFCPPPCIYLLGDGWRKKQEQMVRDGESEQGSQLCAFIGIGNSDQEMQQLDFNGKASTQYSYCAAKTLYISDSDKRKHFMLSVKMFYGNGEDIGVFHSKRIKVISKPSKKKQSIKNADLCIASGSRVALFNRLRSQTVSTRYLHVENGNFHASSTQWGAFTIHLLDDTESESEEFTVRDGYIHYGSTVKLVCSVTGMALPRLIIRKVDKQTALLDADDPVSQLHKCAFYMKDTERMYLCLSQERIIQFQATPCPKEPNKEMINDGASWTIISTDKAEYTFYEGMSPVRSTVTPVPVVHSLQVNGGGDVAMLELTGENFTPTLKVWFGEVEAETMYRCQENMLCVVPDISAFREGWQWVRQPTQVPVSLVRNDGIIYSTGLTFTYTPEPGPRTHCPAVEEILRPPEMRRDDPRHPSHMYSNADHPMQ

>Limulus polyphemus 1 (horseshoe crab; Arthropoda; Xiphosura)

MIEIKRERFESCQEVDVGVMGNNHAYMTEDQYGYTLGSGYEPPPNSTLPLTEATLLSHYSTGNTGPSSPGVNNASVYSTSRGGGICQQGSIMMDISDSSVMNGDVVGHHASDGTFQHLQSRVRANDHSGRGPYDGHPVDLSNQRPDSHLSHMNLGSYVGTHYRSMSQNRSHFENEHSVSENESSMQMLSSSLSVSEKPTIDHQQVNHLPPFSGQNPLGGSTLGSRTHPSPSPLPTPPPIDDDGHRSRRRDQRLTREAMKNYLKERGDMVLVILHAKVAQKSYGNEKRFFCPPPCIYLLGDGWRKKQQQMVRDGENDQSAQLCAFIGIGNSDQEMQQLDFNGKVWKNYCAAKTLYISDSDKRKHFMLSVKMFYGNGEDIGVFHSKRIKVISKPSKKKQSLKNADLCIASGTKIALFNRLRSQTVSTRYLHVENGNFHASSTQWGAFTIHLLDDNESESEEFTVRDGYIHYGSTVKLVCSVTGMALPRLVVRKVDKQTALLDASDPVSQLHKCSFFMKDTERMYLCLSQERIIQFQATPCPKEPFKEMINDGASWTIISTDKAEYTFYEGMGPVHSPVTPVPVVFSLHLNGGGDVAMLELTGENFTPVLKVWFGDVEAETMYRCQESMLCVVPDISAFREGWQWVHHPTQVPVSLVRNDGIIYATGLTFTYTPEPGPRPHCRTVEEILRPAGSHTHPPDGEEPGPLISTYTHVSSGL

>Centruroides sculpturatus (scorpion; Arthropoda; Scorpiones)

MNDVGIFQTEEPYGYPVTHNYQEENRNFDVSSSVQIPQYSVVISSPITTPSSNASVYSLPIQDSNSHRQNMMMELGQSQHSQQSVNHMQINGDVLGHHNSNSLLHSQTQHNASSEHRGSGYESSPVDLSSHRSVGRFGPVDMDAHLGAASIAHYRNLSQEDNSFENGLRNAVPVSMSDTESSMQLISGSMTSHDKVNGDQHSLGSLNSSNALSVPMLAHGRGHAPPSPLPTPSPPVDDRHRNKRKDQRLTREAMKKYLRERGDMILVILHAKVAQKSYGNEKRFFCPPPCIYLLGDGWRKKQEQMIRDGEIEQGAQLCAFIGIGNSDQEMQQLDFNGKNYCAAKTLYISDSDKRKHFMLSVKMFYGNGEDIGVFHSKRIKVISKPSKKKQSLKNADLCIASGTKVALFNRLRSQTVSTRYLHVENGNFHASSTQWGAFTIHLLDDNESESEEFTVRDGYIHYGSTVKLVCSVTGMALPRLIIRKVDKQTALLDADDPVSQLHKCAFYMKDTERMYLCLSQERIIQFHATPCPKELNKEMINDGASWTIISTDKAEYTFYEGMGAVKTPVTPVPVVHSLHLNGGGDVAMLELAGENFTPNLKVWFGDVEAETMYRCQEGMLCVVPDISAFREGWQWVRQPTQVPVSLVRSDGIIYATGLTFTYTPEPGPRPHCPGVEEILRPAGLHQVDENQSRVTPNTVPVAPPHGYPEAHM

>Euperipatoides kanangrensis (velvet worm; Onychophora)

MNEVYISQGQYGYTVGANFQRDNEILYGLNGIPTSLQYPLQASSHNDQQTQFFLQDGSSEQQQQQHNLSDMNRTQQHAHINGHIQPNSYDNPVDLSSHRSSGGGNGRGHVEINGHLNKHPHHHGTVYRDPPPAHNGTYKQHNGLNIPDHIESSQQILPGSLGPSDKVNGDLVSLATTSPLSISTMATTIQTPPSPLPTPSPPVEDRRHGHKDQRLTREAMKRYLRDRGDQILVILHAKVAQKSYGNEKRFFCPPPCIYLFGDGWRRKKEQMERDGETEQGSQLCAFIGIGNSDQDMQQLDLNGKNYCAAKTLYISDSDKRKHFMLSVKMFYGNGEDIGVFHSKRIKVISKPSKKKQSLKNADLCIASGTKVALFNRLRSQTVSTRYLHVENGNFHASSTQWGAFTIHLLDDNESESEEFTVRDGYIHYGSTVKLVCSVTGMALPRLVIRKVDKQTALLDADDPVSQLHKCAFYMKDTERMYLCLSQERIIQFQATPCPKEPNKEMINDGASWTIISTDKAEYTFFEGMGPVKAPVTPVPVVHSLHLNGGGDVAMLELSGENFTPSLRVWFGDVEAETMYRCEESMLCVVPDISAFRGGWQWVRQPTQVPVSLVRNDGIIYATGLTFTYTPEPGPRPHCTAADQILRTTSTVSTVSYNSM

>Platynereis dumerilii (clam worm; Annelida; Phyllodocida)

MNTHQQIYVSQGQYGYTIGADLSRDEQLWATVNGQEHVNLQNNNSYLHNDYLTEQQQTQDCHASGHLPHDLGGGGVMSRPPPHLHIRNNNNPMASENPVDLSSRHVGTPKQQQQQQRNNGDINNLKRKSPPEFDQHHHRHHQEHPSQHSHPQQHPSQQQLYHHNGINNTLRERLHTEPGNHFPGTLTPPDKLNGDHNAHHHHIQHHPSHPHANPHHPHHHGPPPHLAGHMAPHGIPLSTSSPLAISALATPMQTPPSPLPTPSPPHRPGDIENYHHRSINSQYPGQRLTREGMRNYLRDRGDQVLVILHAKVAQKSYGNEKRFFCPPPCIYLFGNGWKRKREQMERDGASDQESTVCAFMGIGNSDQEMVQLNLEGKHYCAAKTLYISDSDKRKHFMLTVKMFYGNGQDIGVFNSKRIKVISKPSKKKQSLKNADLCIASGTKVALFNRLRSQTVSTRYLHVEGGNFHASSTQWGAFTIHLLDDDEGESEEFTVRDGYIHYGMTVKLVCSVTGMALPRLIVRKVDKQTVLLDADEPVSQLHKCAFYMKDTERMYLCLSQERIIQFQATPCPKEPNKEMINDGAAWTIISTDKAEYTFFEGMGPVKAPVTPVPVVSSLQLNGGGDVAMLELSGENFMPSLKVWFGDVEAETMFRCEVSMLCVVPDISAFRSSWRWVRQPLQVPVTLVRNDGIIYATGLTFTYTPEPGPNHQSAAAACVMGHPTPNDHQDSQHALETLT

>Octopus bimaculoides (octopus; Mollusca; Octopoda)

MNEKDHMNTSQNSFLYAFGNSTETGAQLMYTSSNHGLPGSVCTSEAGYIVYTTAAPGMESVTDSSGLLPQPHLQEVITEHLDNHLVHPRTTLINGRMAQNGFDNPMDLSNGKVVQVIDMKKEDSRQYDHNQAATNYHHNGVTVGIPESQHGASEHLMPAGSLTPPDKISGDSISMANIRPLGISTLTNTIKTPPSPMPTPSPPINRITGDIDHSDSRITHPFSRSHWTTDVLENRHVGQYQDQRLTREAMRKYLRDRGDQVLVILHAKVAQKSYGNEKRFFCPPPCIYLFNNGWKRKKEQLERDGATEQESLVCAFMGIGNSDQDMVQLNLEGKNYCAAKTLYISDSDKRKHFMLSVKMFYGNSQDIGMFNSKRIKVISKPSKKKQSLKNADLCIASGTKVALFNRLRSQTVSTRYLHVEDGNFHASSTQWGAFTIHLLDDNESESEEFTVRDGYIHYGSTIKLVCSVTGMALPRLIIRKVDKQTALLDADDPVSQLHKCAFYMKDTERMYLCLSQERIIQFQATPCPKEPNKEMINDGASWTIISTDKAEYTFFEGMGPVKATVTPVPVVSSLHLNGGGDVAMLELTGENFTPILKVWFGDVEAETMYRCEESMLCVVPDISAFRAGWRWVRQPLQVPVTLVRNDGIIYATGLTFTYTPEPGPRQHCNAVDRVLRANSDPPPPSSVNYSAPPM

>Crassostrea gigas (oyster; Mollusca; Ostreoida)

MNEQHLYVSQNGYTYSVGPTQQSSHLMSCSQPQQNGSHTGHGFMHHNAGYTLHPPDRQYGGRADPGPSSRADLTGHMAHGGYENPMDLSSNKPGNPGRLVKEEGHHHGYLAAMGTTPVSVGIPDHVHNPSHIVAGSLTPPEKINGDPGAMATSSPLSITTMTQAIPAPPSPISTPSPLYASNSYVDRGYQDQRLTKEAMRNYLKDRGDQVLVILHAKVAQKSYGNEKRFFCPPPCIYLFGSGWKRKKEAIEAEGGTEQDSTTCAFMGIGNSDQEMVQLNLEGKNYCAAKTLYISDSDKRKHFMLTVKMFFGNGQDIGVFNSKRIKVISKPSKKKQSLKNADLCIASGTKVALFNRLRSQTVSTRYLHVENGNFHASSTQWGAFTIHLLDDNESESEEFTVRDGYIHYGSTIKLVCSVTGMALPRLIIRKVDKQTAILDADDPVSQLHKCAFYMKDSERMYLCLSQERIIQFQATPCPKEPNKEMINDGASWTIISTDKAEYTFFEGMGPVKSPVSPVPVVNSLHLNGGGDVAMLELSGEFLAPNLKVWFGEVEAETMFRCEESMFCVVPDISAFRAGWRWVRQPLQVPVLLVRSDGIIYSTGLTFTYTPEPGPRAHSREVDRIIQPGVSSPDSTSTANFSNPL

>Lottia gigantea (limpet; Mollusca; Docoglossa)

MNEHHLFGSQDGYGVNLDGANLSHHGVHYAIHSNGNFVSSQQGRGIKRTNHELPPHLNTVHYLINGHMAQAGVENPVDLSNGRISMTGLSSDSDNQYERHSDDRQSSQQQQSQYRHSGVRVGIPDQSHDATSHLFTGSLTPPEKPNGDLVPMSTTSPLSITTIGPPMQAPPSPLPTPSPPMRHPPDGSGLRVPQCDAPHLSMRKDQRLTKEAMRKYLRDRSDQILVILHAKVAQKSYGNEKRFFCPPPCIYLFGKGWKRKHDQMEEEGSTKDEAQVCAFMGIGNSDQEMVQLHLEDKDYCAAKTLYISDSDKRKHFMLSVKMFYGNGQDIGLFLGKRIKVISKPSKKKQSLKNAELCIASGTKVALFNRLRSQTVSTRYLHVENYNGKCNFHASSTQWGAFTIHLLDDNEGESEEFTVRDGYIHYGSTVKLVCSVTGMALPRLVIRKVDKQTALLDADDPVSQLHKCAFFLKDTERMYLCLSQERIIQFQATPCPKEPNKEMINDGASWTIISTDKAEYTFYEGMGPVKNSLTPVPVVNSLHLNGGGDVAMLELNGENFNPSLKVWFGDVEAETMFRAEDSMLCVVPDIAAFRPGWKWVRQPLQAPVSLVRLDGVIYATGLTFTYTPEPGPRQHCKDMDRIVGRTSTASPDSTSHTTL

>Notospermus geniculatus (ribbon worm; Nemertea; Heteronemertea)

MRSVDKIMNEQEIYVSQGQYGYTIGVNQQRDNRHLYPNNVMPCSLQAADTNGLLPENTKTMQNGNMSDQLVSHNRMHPNGRMAVQYDNPIDLSNRLEGATQHAQGRDLNGYGKIPRYEHHVDTTSYRHAHSQNQSGQNKVTSSMNMVGIADQSHESGNAHNHMIPGSLTPPDKVNGDMVPLPTTSPLSISTMATSIQTPPSPMPTPSPPLSHHRSEHDTRHHNGQFQDQRLTRDAMRKYLRDRGDQVLVILHAKVAQKSYGNEKRFFCPPPCIYLFGNGWKRKKDQMERDGAAEQESQVCAFMGIGNSDQEMVQLNLDGKNYCAAKTLYISDSDKRKHFMLSVKMFYGNGQDIGVFNSKRIKVISKPSKKKQSLKNADLCIASGTKVALFNRLRSQTVSTRYLHVEGGNFHASSTQWGAFTIHLLDDNESESEEFTVRDGYIHYGSTVKLVCSVTGMALPRLIIRKVDKQTALLDADDPVSQLHKCAFFMKDTERMYLCLSQERIIQFQATPCPKEPNKEMINDGASWTIISTDKAEYTFFEGMGPVKSPVTPVPVVNSLHLNGGGDVAMLELSGENFTPTLRVWFGDVEAETMYRCEESMLCVVPDISAFRAGWRWVRQPLQVPVSLVRNDGVIYATGLTFTYTPEPGPRPHCRDADQIMRPGGTVQALQSPDSSSTHFVPVPNQM

>Malacobdella grossa (ribbon worm; Nemertea; Bdellonemertea)

MNCELYVPQGSYGGYTIGANLQRDIPLYQHYNNYLNQQQQPQQQPARPENNNYRQQIYGNGYNANQQLQQPTGQQSSTDMVDSPFCLSDNANKQNGSHFEAKRNANGNPYNAKMASERLSIPSALTRALHYDNPLDLTNRLDEVREDARHDCAKIARLAYDDDISCARKNSPSALTTLHAGLPDSSHDGSGIAGSMTPPDGGKGNDLDLQSSMATTSPLSIQTIANSIQTPPSPVQTPSPPPPLLRSCRNENDGHNRNGLQMAALPRGSHYGHYSDQKLTREAMRKYLKERNDQILVILHAKVAQKSYGNEKRFFCPPPCIYLFGRGWKRKRELMTSDGATDQESLVCAFMGIGNSDQEMVQLNLEGKNYCAAKTLYISDSDKRKHFMLTVKMFYGNGQDIGVFHGKRIKVISKPSKKKQSLKNADLCIASGTKVALFNRLRSQTVSTRYLHVDGGNFHASSTQWGAFTIHLLDDNESESEEFTVRDGYIHYGATVKLVCSVTGMALPRLIVRKVDKQTALLDADDPVSQLHKCSFHMKDSERMYLCLSQERIIQFQATPCPKEPNKEMINDGASWTIISTDKAEYNFYEGMGPVKAPVTPVPVVNSLHLNGGGDVAMLELTGENFSPNLRVWFKDVEAETMYRCVDTMLCVVPDISAFRAGWRWVMEPLQVPVSLVRSDGVIYSTGLTFTYTPEPGPRPTWRDENSLHANTTLCNGGLTNQHF

>Lingula anatina (brachiopod; Brachiopoda; Lingulida)

MNEAEIYISQGRYGYTIGANLNRDDHIHSYLDSQQQQHVACANIIAEELIYQATDSKFTEDGYVKMNRETASQNGRRGGYENPMDLSRRTEMNGHVGHSYEQRDQSYINHNSVSSSVSVAIPDQSHEASSSAHMIPGNLTPPDKVNGEMVPMATASPLSISTMATSIHTPPSPLPTPSPPVRHPGDGERGDMRHINGHFSGQRLNREAMKKYLRDRGDQTLVILHAKVAQKSYGNEKRFFCPPPCIYLFGSGWKRKKEQIEKEGGSEQDSAVCAFMGIGNSDQEMVQLNLDGKHYCAAKTLFISDSDKRKHFMLTVKMFYGNGQDVGVFHSKRIKVISKPSKKKQSLKNADLCIASGTKVALFNRLRSQTVSTRYLHVENGNFHASSTQWGAFTIHLLDDNESESEEFTVRDGYIHYGATVKLVCSVTGMALPRLVIRKVDKQTALLDADDPVSQLHKCAFYMKDTERMYLCLSQERIIQFQATPCPKEPNKEMINDGASWTIISTDKAEYTFYEGMGPVKAPVTPVPLVHNLYLNGGGDVAMLELSGENFTPHLKVWFGDVEAETMYRCEEGMLCVVPDISAFRSGWKYVRQPLQVPVSLVREDGVIYATGLTFTYTPEPGPRPHCAAIDETMRPGASVIQTTDSVHETQHQQYSNSSSTAVS

>Phoronis australis (phoronid worm; Phoronida)

MTTMATQGQYRYSIQRDTYASHPAFDQDAFCPDPADFECFRTGTLDVSDVRQTHLSVVDSMALSSRQHDNRPMDLSSRGQHHHHHHHHQGVLSSVHHRALKDHYTAEQAAMFYGNVTSSVTVGIPDQSHESASHLIAGSLTPPDKVNGDVVSMATASPLSIGTMAPSIQTPPSPLPTPSPPLSRTEMDSGYNGHFQEKRLTKDAMKKYLRERCDQTLVVLHAKVAQKSYGNEKRFFCPPPCIYLFGSGWKRKKEEMEREGRSEQESQVCAFMGIGNSDLEMVQLNLDGKNYCAAKTLYISDQDKRKHFMLSVKMFFGNGHDLGVFNGKRIKVISKPSKKKQSLKNADLCIASGTKVALFNRLRSQTVSTRYLHVEDGNFHASSTQWGAFTIHLLDDNESESEEFTVRDGYIHYGSTIKLVCSVTGMALPRLIIRKVDKQTALLDADDPVSQLHKCAFYMKDTERMYLCLSQERIIQFQATPCPKEANKEMINDGASWTIISTDKAEYKFYEGMGPVQNPVTPVPIVHQLHLNGGGDVAMLELSGESFTAKLKCWFGDVEAETMYRCEESMLCVVPDISQFRAGWRYVRQPLQVPVSLVRNDGVIYATGMTFTYTPEPGSRPRCPASEAMLHGSVTMASPDSTYNNCGS

>Xenoturbella bocki (paradox worm; Xenacoelomorpha)

MSTHLVPTTTFDYGFSTNHSQLPSSHYAAVYHGLPSTERIPLDRHSTDPPGDGELTPRFAEVFHTVPTTGSDVYGSQKLINPQSCGMPQLYPSNINSHLLTNDNHRRMSCDNNGSHMTVPQDGMYDDRTNGHELKTSPAVVAGAVAMETSAMTRKQHVFRAPRHLVLPVATTYYESPPTTKKSRTVEYSMRSPLEGATNTSPYGMMGHAPQGLGGHSPSLGGHSPALGGHSPVLGEPADGDLPVLKSSELSSMGAKRYSAPLNLTVHDKCKDVRVLGRLTPPDKQHVNNDVGALLSPSALSPHSSDASDTESTQPPSQTIPLTPPIDDTQYSCSKHLTREAMQRYLNDRPDCTLLILHAKVAQKSYGSEKRFFCPPPCVYLLSSGWKIKRDHTAESQICAYMGIGNSDQEPQLLNLDNKTYCAAKTLYISDSDKRKHFMLSVKMAMSSSGLGADIGTFMSKRIKVISKPSKKKQSLKNTDLCIASGTKVALFNRLRSQTVSTRYLHVEDGNFHASSTQWGAFYIHLLDDAESESEEFTVREGYIHYGSTVKLVCSVTGMALPRLIIRKVDKQTALLDADDPVSQLHKCAFFMKDTERMYLCLSQERIIQFQATPCPKEPKKEMINDGASWTIISTDSAEYSFYEGMGPVTRSVTPVPIVHSLQLNGGGDVAMLELSGESFSPAVKVWFGDVEAETMFRCTESLLCVVPDISAFRGEWRWVRQPTQVPVTLVREDGIIYSTGLTFTYTPEPGPRHGSITDTLRLHTTANTNTETTTTDHYINPCNSYPGL
