# Supplementary material for: Evolutionary emergence of Hairless as a novel component of the Notch signaling pathway
Source: eLife. 2019 Sep 23;8:e48115. doi: 10.7554/eLife.48115 (PMC6777938; doi:10.7554/eLife.48115)
Supplement: Supplementary file 5. — Characteristic domains and motifs are colored as follows: Su(H)-binding domain (SBD), orange; CtBP recruitment motifs, red. [file elife-48115-supp5.docx]

**Supplementary file 5.** **Full-length S-CAP sequences**

>Strigamia maritima (centipede; Geophilomorpha)

MPNPNCETCESSQNNKRKCENNANVQSGNKKRSTDPNECQQPAQSAHDFLKKLVHEQKLHAPTLFNNTNELKPENNRTGSSENDTNGKLSFYLDGRFILELEHKPGGKRRNGGWIQTKGITWMPRPEEEKLINPIKQELKESETSRMKLKNLKRFSSQRETTNACSVVLFNFRINRRRDFKHFPRLNAYYLRKAARHPFSIDLFFFSKSEKCKTAPPSTKLVKKRTKRLNDLFRPPEFVNSTADNVKRWFDEVGSVKVKCEPDNASLPLPEWYLDPSKSQIPVNEMIGNPRPYLPAAPTHYDDKESNVNSMSEPLDLRTNVVRETKRDEWMKREAKEHPGNAFYVGLIPFPLPGHGEYSAPSAVQPWPCVSCPVVAYAPTVVNSYYYCCCFLPGCRSNCLCKTEGKQLTSRSSEIPKECDCATDLTNACNKKFPIPVAVKSDHQTPGKKTDVGDGCSHSMLKDLLLMPSSEKDCHCPQTSNP

>Sigmoria latior munda (millipede; Arthropoda; Polydesmida)

MPHSVDPEQDDDKFTQAHVDREGDAASSVGVMANCVRSNHNNLGSAEYQGSSSSSSGGGGSSSSSSSGGAASDDDEGGECSLDSYQSDKNSVHSPNTNRCHPSLQRASPGLTNGIEVKPCRSVVDDELRDRLTNANELSESTNAASAEKKDDSLGGYLRFFLDGRCVLELRHDCSNEGQPKQWVQSYGKTYRSSIASVPTSCTPAWQPQESSNHDVSEDIRVNSPSVTLSPNKKKPYNNFYLTFFARNRREPNPNVLPSCVLRKAARRPGEVVSKEFCCPHEKMCCKRHPLRQRTTTLVQKSQRLNLLIDGLAHEYSGESLPGSKNSRKSGSLGGLLLGALIQKKSSDSSKNSHVQALTKRISSPAVVGQEDHDEVEKGGANKPLPEWYFDPSKATIPVDEMIGNPRPSLLSSQSTLVSNKRPSDDSYPSHSEAKKQAKEDTDTVDVDSKNQPFSSHCCVPHSKSPSLEKTNVAASPFKVESDVCEDTTPASAVNATGKNSETVQRKKKKGGGYGRGRTIGEAVRTFVISEILNLGGDVRTNYVPRGVFTEVASRYKIDATTTYRWWLKYCNGIRMAASGNINQPDTSHKGFLSNRVTAVPVMVPDHKTRADHFYKEVKSLHNPHILESSTLKRDHMALMASGASMSHGGRYSPRSDDSASSGASLVDPTALYVSSLTLAPPTGVWPAGVPLGSLPNSQLPFQTMRLPLPLSVCVPYLWNCSTEQPNVNGISGGKQNHLESKGSPIINSDEPLDLTVPKKEKSDGSSLAHCSKIENLKVLNTGLGTYSECQSDQFILRTLLSTHGQPTTAGSNNLVDKEASYSIGKPFAKSLAQRNMKPKHYPFLSLQIGSFEIHSNLDTSQSCDRDHFKEIPNKFKVIFTQRKFVYEFETNAEMHLPSDDGNQKESPPSVKRMACIVVPFNIVEAFWCYGATIVLRVVSQPFMFLGRKLKQVGCVPSLTVYETKKSVDLTGGQLSMAHLHKVTLIKPHLIEQLKIYLVLFDVRFRNLISPVAHSLVSTEDRAAEHTETND

>Nephila clavipes (spider; Araneae)

MDKCSTDAEFDSYWDYEAKNKNRNRIRVGRQYQATIPPLLKPGQKDGRKSEELETLKWKPDQLSDQALEEYLSMAKGIGLFSKSLDSPKPSEKSDNSLQSAIKGLSEFVTSHHPCHHDDGCKVARPSSSGESSSKSTATSDWSPSEAQLFAQALEACGKNFGAIKKEYLPWKPIKSIIEYYYEGKNEKLEASSEAETSGKISPKKELGSPASYKEEKDFIKEEVLPDEAVPEGSIPEDPPKDAINFLENTRVTDPCRATPVELKMSVNENDIVTASSTVASLKFFLGGRLVLKLNAQQDSGSGTKCQWVQSNDLPKHSNNIKKDRQKKKCIQDESFVINPDVHKLKKKTEELSSASNELTDCDPSAVKKAKLKSEYDFPLCSSSWTPPVADGQESIEVDDASSKTSEGYISPVLSNSRFSKADAKLDSVSNFNSVGDPKCDSDMCWVKSEMKGCNISSDHCNDSASKNICMLGGKNSPNHMSDVSPYKQEVLNHKSALSHKTCVKSLCYQPHTSHSNRSLSKQWSKRSNMTYPMRTSPCSLSPISVVDLKSPSKETPLDLSSKLDNVLNDIDHHNSQSAHKTKVQVDNAEKIMPSESESRPASSEFSPQSSNHGSLAQSIDSPYKDTINSHKCSEDTASDHQQCEKCLRQEDSDFSSGKDSENSCQLPYTYCNLPYQCALPKCTKDCLLEEHLCNKSDAEKSQAGSPGYIINDVDENFCKKNPAWCSRSGASYYPYYSQCYPYYLSYGVAPSHVSEPTIAEVPLDLSSTNDKVKENSSETEKLPEEVNVMNSDDTDAVERGMLYQLLKNKTSK

>Parasteatoda tepidariorum (spider; Araneae)

MDKCSTDAEFDSYWDNEAKNQNRNRIRVGRQYQATIPPLLKPGEKDGRKLEDLETLKWKPDQLSDQKLEEYMSMAKGISLFSKSVSSSRSPEKSDNSLQSAIKGLTEFVASHHPCHHDDGCRQVLKPSSSSTESVSKVNTNTWSQSEAQLFAQALEACGKNFSAIKKEFLPWKPVRSIIEYYYQIKNEKAEETEPTDKVIPKAEEEPIPSCSHEEKPCVKEEPVKVEEPQVKTVNTEDPPKDSINFLDHSRVTDPCSAASETSLPQDVPATSTVGSLKFFLGGRLVLKLNAQQDGGSGNKCQWVQSNDLPKHSNHNKKDKHKKKFAPYSYSSSGTQKPLKKGDDTSAVPDCDPSGIKKPRLKEYETSENSALGLLLCSSSWTPPVADGQESIDVDDTSSKTSEGYISPILSNNSRTSKIDTIKHDFASNPNTSNELGEQKCDSDPTWVKTDNCIISDSASKNASVLSGKNSPNQCYSSPMKKEVLNHKSALSHGRSVKSPCYQSRSSHSSSPLLKQRNIKWSNIPYPLKSSPSSLSPVYIDLRSSTKESAIDLSSKSDNGLNNADHYVKQTHSNERSECNECILCESDSRPDSEFSPQSSNFTPSAESPRKEPSCTACNCCEGVSTDQLPFDNSGRKEDETMTNKESDDNSQLSYTYCNLPYHCALPKCTKDYTLEEHLCNKSSINKNQVDCPEYIINDIDENMCEKNPNWCSKASYIPYYSHCYPYYVSYGVTSRNSAESTDTEVSEAPLDLSSTNETKPEKLPNVEKISENSSEEVSQNAEGGRGMLYQLLKNKSK

>Stegodyphus mimosarum (spider; Araneae)

MDKCSSDAEFDSYWDYEAKNKNRNRIRVGRQYQATVPPLLKPGEKDGRKCEELETLKWKPGQLSEQEVEQYLSMAKGIGLFAKAVDSPKIPEKSDSSLQSAIKGLSEFVASHHPCHHDDGCQVPKPSSSGETDSKVNATNDWSPSEAQLFAQALEACGKNFCAIKKDFLPWKPVRSIIEYYYHGKGEKTENLADDSDTKLPEKKEEDEPVASCSHTEKSSESEMLPSDEPQSEDLNVEEPTEPTKESVLFLENSRVIDHCQAVPELKMSVTEDGMPASSTVGSLKFFLGGRLVLKLNARQDSGSGSKCQWVQSNDLPKHSNTTKKVKHKKKSMQDGSFPASDIFKQQKKSEELCISNRVSDYDTSVVKKPKLKLEHISHLDKQELLLCSSSWMPPVADGHESVEIDDGSSKTSEGNGNVSPVLSNPRSIKADVKTDFASNFVNELGEQNCDSDPCWVKLESQCGNVATESSFVDNSASKCVSYVGENNSPNQFIDDSSFKQEVLNMSALSHNKAHTKNQCYQPLTSHSNSPLLKQWSKSPNPVKTSPSSLSPVSLIDTKSPVKDIPLDLSSKLENSINSTDHHDMMDIHKPTVKVECSNQSFPSETETSPVNCEFSPQSSKLGSHVQSNESWRKETKNNVYQNSEDKADPLQYVKEENNEYSPKKRCGDPSQLPYYCNLPYHCASPKCTKDYSLEEHLCNKSNVEKNQENDPSYILQNVDENVCKKSPWCSGTVASYLQLYPPCYRSYYYSYGMAPSPASEPTIAEVPLDLSSAGESIAEKIAMPENLPEDITDVSQTNTEAVERGMLYQLLKNKTSK

>Achipteria coleoptrata (mite; Oribatida)

MDVSNSDADFDSYWEEEARNPNRNRIRIGRQYQATVPPILKQGESDGRKLEELETLRWKPDNELTDQQLDQYLSVAKAVSLFTRAIDNNYSQSNVNSDSHIENDETNETNSNSNQNTNRDKTENKNDKNKDNTLSPGSTSTVKDNRIQSALKGLSDFVSSHHPNHRDIGCRTPLLPETTANSAESESANSLTNLLSAEWNSNEIKLFSRALEVCGKNFGAIKKDFLPWKSVTSIIERYYLGIGRDGPNSGIKHEEQNNINQSGNSQNNKQESVPNTFVSDILSSVSKFMTSYTDAKPELNECQRTQSSQMSSEDIKDCDLKNNFNSEDSKIASVSGQEVKPLKAKPILPTAIQESNNSVSTVGSLKFYLDGQLVLKLNAQQEMMGQKCQWVESSDTAKKARLLHKTKKRLLNERHDSDVTNHSNHKNSNSEELDDGSNESSDDDSMESNESALVPSPSAFIAKKAKVKVENSSHKPLSSPTSLPMKDSKLDVSNRNVKIECSNSSVKKEVHLSPNSNHCEDEHKKRFSSEHTNERTNSQNCLENKWYHTIVEKSSLQPPKAHSMTSSGPLYRHSRYDSVQSSLGSSSSTSSPSSSSSSGTGVPLMSSNAIPVDLTRKSTNYSPFEAKSSSLSSSSTPDFGNYPERDKNSKVLSSPSPPRLQPPFVFPYPAYLPLSPTQSQNKIKREKEVKSSNNLCQKAVEGRPLVKSSPPTSPSLMAPSSTPPLPSALPSPSQLAWIQNSFAPYLPIYEQYYRYYYGYGMPSPNHSPGERMSSSQTPKENEANSKERAKCLTDAVLVDGDDANCGS

>Sarcoptes scabiei (mite; Astigmata)

MLDIDFDSYWDEEARNPNRNRIRIGRQYQAQCPALLKSGQNDGRKLEDLETLTWNPNNPLNIHQLNQYFSIAKSIGLFIQAIDTCQDGIDEIECLEEEKCSTRPFQILDSGTKCILEAKPDRTVQSSNDNNNVINTSVSIPEKHQSDKVTTRQRIQHHHKSNHKEDSVEVTSQSSSNSSSSSSTTSNSLSTSPLKQTSNTRIQSIAKGLPAFILSHHSKNMREEKITDDDDYESKNNKGKNDDELQDSKCKFKSTTAEQAINLGSVEKYDVKKLANLLMGKWNATEAKVFAQAFNQCGKNFMAIKKDYLPWKSIRSIIEYYYLTCDKEKEELRKQRRNRAKSNRNCFSLNENRTNTTKPPSSSDNSDEINYESAKKFSTDIDGSNPKFNISQNSNQKSNDDRTRLSPNKQSNLSEQDLFNLMLMKGGLKNGNINLDAIADFTNSRFKFNNTNNNCNINGGNSAFIQSSSESKNSFVPGQEVRPVKAKPIFSQTDPSSSQLNEADSSKTNLGSLNLYLHGELVLRLNAQQQDSGQKWVESNEIQSSFKDDADDFSCGGADVSASDDDSLTSNESSSLVASSPSSSSTIATSSAKKSRVKLEHQNSNFPSNSQNSSISSPVSSHRNKNESNFNGGALNNPFPNTSQSLINSISALAAASSFLENPLSELEIKKRLFEYCQMGMSVEELQLLLASNLFPDPPKAHSNTPCLAATVPHIGTNVTDKTTTQNVSEKDSVYRSSPVKEHQRSSKSSSKQSKIVSNDLDNADNGYTNGFGPIDLTRRKSSINHNPFNSISNEMGSKNHSPKSKQSFSASTSNNLLNRSNCSLFSLPTLSSSSPSSLSSSTLSNPNGTGRNSTIVSPAKFSSNSHSSKSMKNNYNSKKKSSSSSSSSSISIAETNPF

>Metaseiulus occidentalis (mite; Mesostigmata)

MDSNNDVDFDAYWEEESRNKYRNRIRVGRQYQASVPPLLRPGESDDRQLEDLETQYWKPSLDDEAIHEYLSMAKAVSVFSCSLESQSHDNLQSAIRGLTEFVMKHHDPCHRDAGCRMSVKSSWTNKEAELLACALERCENANRKKMAEEYDESGSDDADGVDETSDIEADCQPSSTSALELPIDVNPPSEIKPVPARVIGRKTDSPVSDSGSPTGPAQATGTAAGGATPVSAKSRADGANTSQQLQDPSAAGQGSLKFYLKGQLILKLNAHQERKTWVEDPDNPAASSGQFGGVAGALSGGQNRRKAGRKANRSSTTSGPISWLDRGSPSLESTSSLDSHNSTSTSATTPTCAAATPTPSTTAATSTAATNALPAGGNNGGGGGGSSSSSSSSCSSSSSSAASSAANPVSQQPAPLDLSSQQS

>Dinothrombium tinctorium (mite; Trombidiformes)

MDVSTSDADFDSYWEEEARNPNRNRIRIGRQYQATVPPLLRPGESDGRKLEDLETLKWKPENDLSDQQLDQYLSLAKAVALFARAITNNSSANQTDSESAHSNGNSGNNTNSNSENQADEEQNSTSQEDKKNAENTDSFVSQPSDHLQCALKGLSDFVTSHHPFSRDAGCKVPINSSDKSATSSSQSNSENFTNSTKLNWTSDESDIFAKALDACGKNFSAIKKDFLPWKSVQSIIAYYYLGLNRTKLDTNCSDKNIKAFSSNDEKDCTTPKMSKDCSALINVATTSTASSPNSPTFSLSPIMNTACKDYSSKKHVNGVSNSLIECDSNSKQNEANCVNLEIKALKAKPVLPSNDDLSCNMSNLGSLKFYMDGQLVLKLNAKQEVTGRKCQWVESQDTPKFSRPVRKGNKKLLLEKSESDSKIGHVHNDKCMHQSNEDGEEGSLDSSDEDSLESSESNAVLSPMSVTSRKAKVKVENNCFVPLPSPCGVSGSKDQSLNRSNSTNESPTSVVKKEPLCSPTSSSNQKNDIEAKRKLKVPPWPPDKKKSLFITATDNKWSIAEKNVKPPEAHAASRILNSGSYSSSAVPVDLTRKSSAYSPNGTKSHSSLSSSSSTSGDAEKISSSPAQLNYPTSLTPNSLSSSQKQSKSAKDLKPIEMCQKEKSPKSDKLSAEKTNSVSNASLAWFQSPLVSYLPIYEQYYQYYCRYGFPPLPNTAASCESKSNKTSNH

>Ixodes scapularis (tick; Ixodida)

MPNSNRLLARRPPCEVPLDADFDSYWENESRNNNRNRIRVGRQYQATVPALLRPGESDGRRLEDLETLRWRPESLSDQSIDEYLSMAKAVSLFARAMDKWQAWGEGGPEGCCLQTALRGLSDFVTSHHGCHHDAGCQVGPTLPCHWTPTEASLFARALDECGKNFGAIKKDFLPWKPVKSLIEFYYQGRMPKQESDGQEQEAGCSTSTGASCTSNCSTSKCTANCTSNCISNCIKNCIKKEVKEEPVEEDEEEEREEEKQKGEEPPPEGTAEEAPGVSGPEVKPMRAKPVKAAEEGPASVAPVGSLKFFLGGRLVLKLSAQEGGAWVEAQDTPRLGRPRQPPPDDASDEEEPSPPGSGGGGPPQKGTSRCTSWPETATPAPASSSAGGASASTPEENNTQNSTEEEEAGDESPPLPAPLREASPKCLPPPASAAPVTGAGTLPPPGCAERCRPLSGWRSPRGAVPFLGPLFAAQCCPTTFLPASTADAPLDLSSPVDAKKLPLVPK

>Centruroides sculpturatus (scorpion; Scorpiones)

MFRLMDASNSDADFDSYWEDQSRNRNRNRIRVGRQYQATVPPLLKPGETDGRCCEDLETLKWKPDNDLSDQQIDQYLSMASRAVSLFAKAIDTCQNTGNGPDKTLQTALRGLSDFVTSHHPCHHDAGCQVPSPSCCGDHNCKHSIKNGWTPTEAQLFARALEACGKNFGAIKKEFLPWKPVRSIIEFYYNGKEEKYDNQSARQSQQIANLSENLITGKENISEAHNLKNEDEEDDDDDDEVDKLEDCDCKLEKTDSKSDHDTETQNEKTSKLNQPNNESTSFNLGSLSFFLGGQLVLKLNAQQENGPDNQNCQWVLSQDTPKLPPNRKGRKSKKSCKQGSITSIGKEEIQQTKNEKKQEFCMEEREEGSGVKKAKLKTDCIHSSSMEEASDFCNMPWLPPTETKQERTEKCDDTLYKTETWCHSFSSLCLSQCKNENCTHKDKNCLKNTGLLNSEDNITSTIKTEPGLSPSYIMNSNVSLKEEDSKESVVSMKSYLKALSVPSSSLSSQNWVEHSNIWRNSTKFNEILTKKETWSHSQTLQDSDGGPLDLSIKNSEKNLSKSCEALTSEKSLTVHDSVASDTQMCPFIKSSTCQKRTSVSTSIKDSLSLNDMSQTNMPLDGWSVQVSHGLPYVKGETVKKDCHSSQLLSKGYPLSCEYSRYGMTVTYAPTVFPPVYNCKKDPTNKDKPVNLSKKNENFTCEENNSTRELEVDERCSESGVDLVPWVVAPILSYGQMHSPFCSCCVTYTDSMGMNRLSDQPQGDSETPLDLSSSSSSSSSSSSSSDTTDITDFHKPKNENVAD

>Limulus polyphemus (horseshoe crab; Xiphosura)

MDASSDADFDSYWEDESKSKNRNRIRVGRQYQATVPQLLKPGESDGRQLEDLETLKWKPNNDLSDQQLDQFFSVARAVSFFARAIDKYQHSDKESDKCIQTALRGLTEYVTSHHPCHHDANCQLSQGSSDPQPSGSNSSNDWTYGEAQLFARALEACGKNFGAIKKDFLPWKPVKSIIEYYYQGKEERPNSSHNNLGEPSTSMFSVVEPKKEIIDCDDLGDPHTSPDSGSSEEPDNSIPEERIDEPSDDKQDDETGSHPNLPTTSLTAEVLEVKPMKAKPVTATLDLETSPLGSLQFYFHGKLVLKLNAQQHANTNGHQCQWVPSVDTPKVPFPCSAGQCKKKYVELYEDSRTVDGADLQEVEGGFSKKAKLKNDFAIVWPEESFGAPVHSPQSPALSEWRHRGRATPYDGTLSPFSGVCSDPSDSDISPLQSPTDKCSTKKILGPSELCSVKDDLCIKPEPVSPCSHPTHNIEGRKIPFYSSCHKQDPQKFNTLEEVKRPFSQVNCTDVSQQIECSVPISYPSMLKTVLEATDKTGYKHLLSSSYKLSNVKQEVPLDLSIKSSQPSVNKLHAVETNCQTDGKLNSCSHKQKQLSSSWPDETLSHPPGLNTRRSSKQIKRSSFSSPDVRPSVSSFKSLTSSPVGGISKQHSADNKWTSINAKPLTLPPVISSATNTLHPGSATWTSFSANPDILSPTHTWEKSDATVASLTSYQEQHSGLTYLSMPIYNMAYPYPANCVENVNGVSASTEVNYFSQKNSPIKLKNMEDVTSSPSSKEKEIPAWVTNRPTDLTSYIPLYPHIYSYYPYAYNLSQVSWNGAVTPTEASDAPLDLSSPPSDNIPCSPKQKETIVGSSRAVQVPKDETDKDELSKLSNKSDDFSPSVSLGF
