# Supplementary material for: Evolutionary emergence of Hairless as a novel component of the Notch signaling pathway
Source: eLife. 2019 Sep 23;8:e48115. doi: 10.7554/eLife.48115 (PMC6777938; doi:10.7554/eLife.48115)
Supplement: Supplementary file 6. [file elife-48115-supp6.docx]

**Supplementary file 6.** Sequence data references not cited in the main paper text.

Almada AA, Tarrant AM. 2016. *Vibrio* elicits targeted transcriptional responses from copepod hosts. *FEMS Microbiology Ecology* **92**: fiw072. **DOI:** [10.1093/femsec/fiw072](http://dx.doi.org/10.1093/femsec/fiw072)

Ando T, Matsuda T, Goto K, Hara K, Ito A, Hirata J, Yatomi J, Kajitani R, Okuno M, Yamaguchi K, Kobayashi M, Takano T et al. 2018. Repeated inversions within a *pannier* intron drive diversification of intraspecific colour patterns of ladybird beetles. *Nature Communications* **9**: 3843. **DOI:** [10.1038/s41467-018-06116-1](http://dx.doi.org/10.1038/s41467-018-06116-1)

Anstead CA, Korhonen PK, Young ND, Hall RS, Jex AR, Murali SC, Hughes DS, Lee SF, Perry T, Stroehlein AJ, Ansell BR, Breugelmans B et al. 2015. *Lucilia cuprina* genome unlocks parasitic fly biology to underpin future interventions. *Nature Communications* **6**: 7344. **DOI:** [10.1038/ncomms8344](http://dx.doi.org/10.1038/ncomms8344)

Babb PL, Lahens NF, Correa-Garhwal SM, Nicholson DN, Kim EJ, Hogenesch JB, Kuntner M, Higgins L, Hayashi CY, Agnarsson I, Voight BF. 2017. The *Nephila clavipes* genome highlights the diversity of spider silk genes and their complex expression. *Nature Genetics* **49**: 895-903. **DOI:** [10.1038/ng.3852](http://dx.doi.org/10.1038/ng.3852)

Barreto FS, Pereira RJ, Burton RS. 2015. Hybrid dysfunction and physiological compensation in gene expression. *Molecular Biology and Evolution* **32**: 613-622. **DOI:** [10.1093/molbev/msu321](http://dx.doi.org/10.1093/molbev/msu321)

Barreto FS, Watson ET, Lima TG, Willett CS, Edmands S, Li W, Burton RS. 2018. Genomic signatures of mitonuclear coevolution across populations of *Tigriopus californicus*. *Nature Ecology & Evolution* **2**: 1250-1257. **DOI:** [10.1038/s41559-018-0588-1](http://dx.doi.org/10.1038/s41559-018-0588-1)

Blankers T, Oh KP, Bombarely A, Shaw KL. 2018. The genomic architecture of a rapid island radiation: recombination rate variation, chromosome structure, and genome assembly of the Hawaiian cricket *Laupala*. *Genetics* **209**: 1329-1344. **DOI:** [10.1534/genetics.118.300894](http://dx.doi.org/10.1534/genetics.118.300894)

Brandt A, Schaefer I, Glanz J, Schwander T, Maraun M, Scheu S, Bast J. 2017. Effective purifying selection in ancient asexual oribatid mites. *Nature Communications* **8**: 873. **DOI:** [10.1038/s41467-017-01002-8](http://dx.doi.org/10.1038/s41467-017-01002-8)

Bunnefeld L, Hearn J, Stone GN, Lohse K. 2018. Whole-genome data reveal the complex history of a diverse ecological community. *PNAS* **115**: E6507-E6515. **DOI:** [10.1073/pnas.1800334115](http://dx.doi.org/10.1073/pnas.1800334115)

Carmona-Antoñanzas G, Carmichael SN, Heumann J, Taggart JB, Gharbi K, Bron JE, Bekaert M, Sturm A. 2015. A survey of the ATP-binding cassette (ABC) gene superfamily in the salmon louse (*Lepeophtheirus salmonis*). *PLOS ONE* **10**: e0137394. **DOI:** [10.1371/journal.pone.0137394](http://dx.doi.org/10.1371/journal.pone.0137394)

Chen W, Hasegawa DK, Kaur N, Kliot A, Pinheiro PV, Luan J, Stensmyr MC, Zheng Y, Liu W, Sun H, Xu Y, Luo Y et al. 2016. The draft genome of whitefly *Bemisia tabaci* MEAM1, a global crop pest, provides novel insights into virus transmission, host adaptation, and insecticide resistance. *BMC Biology* **14**: 110. **DOI:** [10.1186/s12915-016-0321-y](http://dx.doi.org/10.1186/s12915-016-0321-y)

Cheng T, Wu J, Wu Y, Chilukuri RV, Huang L, Yamamoto K, Feng L, Li W, Chen Z, Guo H, Liu J, Li S et al. 2017. Genomic adaptation to polyphagy and insecticides in a major East Asian noctuid pest. *Nature Ecology & Evolution* **1**: 1747-1756. **DOI:** [10.1038/s41559-017-0314-4](http://dx.doi.org/10.1038/s41559-017-0314-4)

Christie AE, Chi M, Lameyer TJ, Pascual MG, Shea DN, Stanhope ME, Schulz DJ, Dickinson PS. 2015. Neuropeptidergic signaling in the American lobster *Homarus americanus*: new insights from high-throughput nucleotide sequencing. *PLOS ONE* **10**: e0145964. **DOI:** [10.1371/journal.pone.0145964](http://dx.doi.org/10.1371/journal.pone.0145964)

Colbourne JK, Pfrender ME, Gilbert D, Thomas WK, Tucker A, Oakley TH, Tokishita S, Aerts A, Arnold GJ, Basu MK, Bauer DJ, Caceres CE et al. 2011. The ecoresponsive genome of *Daphnia pulex*. *Science* **331**: 555-561. **DOI:** [10.1126/science.1197761](http://dx.doi.org/10.1126/science.1197761)

Cong Q, Shen J, Li W, Borek D, Otwinowski Z, Grishin NV. 2017. The first complete genomes of Metalmarks and the classification of butterfly families. *Genomics* **109**: 485-493. **DOI:** [10.1016/j.ygeno.2017.07.006](http://dx.doi.org/10.1016/j.ygeno.2017.07.006)

Dong X, Chaisiri K, Xia D, Armstrong SD, Fang Y, Donnelly MJ, Kadowaki T, McGarry JW, Darby AC, Makepeace BL. 2018. Genomes of trombidid mites reveal novel predicted allergens and laterally transferred genes associated with secondary metabolism. *Gigascience* **7**: 1-33. **DOI:** [10.1093/gigascience/giy127](http://dx.doi.org/10.1093/gigascience/giy127)

Eyun SI, Soh HY, Posavi M, Munro JB, Hughes DST, Murali SC, Qu J, Dugan S, Lee SL, Chao H, Dinh H, Han Y et al. 2017. Evolutionary history of chemosensory-related gene families across the Arthropoda. *Molecular Biology and Evolution* **34**: 1838-1862. **DOI:** [10.1093/molbev/msx147](http://dx.doi.org/10.1093/molbev/msx147)

Faddeeva-Vakhrusheva A, Derks MF, Anvar SY, Agamennone V, Suring W, Smit S, van Straalen NM, Roelofs D. 2016. Gene family evolution reflects adaptation to soil environmental stressors in the genome of the collembolan *Orchesella cincta*. *Genome Biology and Evolution* **8**: 2106-2117. **DOI:** [10.1093/gbe/evw134](http://dx.doi.org/10.1093/gbe/evw134)

Francois CM, Duret L, Simon L, Mermillod-Blondin F, Malard F, Konecny-Dupré L, Planel R, Penel S, Douady CJ, Lefébure T. 2016. No evidence that nitrogen limitation influences the elemental composition of isopod transcriptomes and proteomes. *Molecular Biology and Evolution* **33**: 2605-2620. **DOI:** [10.1093/molbev/msw131](http://dx.doi.org/10.1093/molbev/msw131)

Fu X, Li J, Tian Y, Quan W, Zhang S, Liu Q, Liang F, Zhu X, Zhang L, Wang D, Hu J. 2017. Long-read sequence assembly of the firefly *Pyrocoelia pectoralis* genome. *Gigascience* **6**: 1-7. **DOI:** [10.1093/gigascience/gix112](http://dx.doi.org/10.1093/gigascience/gix112)

Fu Y, Yang Y, Zhang H, Farley G, Wang J, Quarles KA, Weng Z, Zamore PD. 2018. The genome of the Hi5 germ cell line from *Trichoplusia ni*, an agricultural pest and novel model for small RNA biology. *eLife* **7**: e31628. **DOI:** [10.7554/eLife.31628](http://dx.doi.org/10.7554/eLife.31628)

Gompert Z, Comeault AA, Farkas TE, Feder JL, Parchman TL, Buerkle CA, Nosil P. 2014. Experimental evidence for ecological selection on genome variation in the wild. *Ecology Letters* **17**: 369-379. **DOI:** [10.1111/ele.12238](http://dx.doi.org/10.1111/ele.12238)

Harrison MC, Jongepier E, Robertson HM, Arning N, Bitard-Feildel T, Chao H, Childers CP, Dinh H, Doddapaneni H, Dugan S, Gowin J, Greiner C et al. 2018. Hemimetabolous genomes reveal molecular basis of termite eusociality. *Nature Ecology & Evolution* **2**: 557-566. **DOI:** [10.1038/s41559-017-0459-1](http://dx.doi.org/10.1038/s41559-017-0459-1)

Holt RA, Subramanian GM, Halpern A, Sutton GG, Charlab R, Nusskern DR, Wincker P, Clark AG, Ribeiro JM, Wides R, Salzberg SL, Loftus B et al. 2002. The genome sequence of the malaria mosquito *Anopheles gambiae*. *Science* **298**: 129-149. **DOI:** [10.1126/science.1076181](http://dx.doi.org/10.1126/science.1076181)

Hunt BJ, Özkaya Ö, Davies NJ, Gaten E, Seear P, Kyriacou CP, Tarling G, Rosato E. 2017. The *Euphausia superba* transcriptome database, SuperbaSE: An online, open resource for researchers. *Ecology and Evolution* **7**: 6060-6077. **DOI:** [10.1002/ece3.3168](http://dx.doi.org/10.1002/ece3.3168)

Husnik F, McCutcheon JP. 2016. Repeated replacement of an intrabacterial symbiont in the tripartite nested mealybug symbiosis. *PNAS* **113**: E5416-24. **DOI:** [10.1073/pnas.1603910113](http://dx.doi.org/10.1073/pnas.1603910113)

Kelley JL, Peyton JT, Fiston-Lavier AS, Teets NM, Yee MC, Johnston JS, Bustamante CD, Lee RE, Denlinger DL. 2014. Compact genome of the Antarctic midge is likely an adaptation to an extreme environment. *Nature Communications* **5**: 4611. **DOI:** [10.1038/ncomms5611](http://dx.doi.org/10.1038/ncomms5611)

Kirkness EF, Haas BJ, Sun W, Braig HR, Perotti MA, Clark JM, Lee SH, Robertson HM, Kennedy RC, Elhaik E, Gerlach D, Kriventseva EV et al. 2010. Genome sequences of the human body louse and its primary endosymbiont provide insights into the permanent parasitic lifestyle. *PNAS* **107**: 12168-12173. **DOI:** [10.1073/pnas.1003379107](http://dx.doi.org/10.1073/pnas.1003379107)

Kono N, Nakamura H, Ito Y, Tomita M, Arakawa K. 2016. Evaluation of the impact of RNA preservation methods of spiders for *de novo* transcriptome assembly. *Molecular Ecology Resources* **16**: 662-672. **DOI:** [10.1111/1755-0998.12485](http://dx.doi.org/10.1111/1755-0998.12485)

Lefébure T, Morvan C, Malard F, François C, Konecny-Dupré L, Guéguen L, Weiss-Gayet M, Seguin-Orlando A, Ermini L, Sarkissian C, Charrier NP, Eme D et al. 2017. Less effective selection leads to larger genomes. *Genome Research* **27**: 1016-1028. **DOI:** [10.1101/gr.212589.116](http://dx.doi.org/10.1101/gr.212589.116)

Li ZQ, Zhang S, Ma Y, Luo JY, Wang CY, Lv LM, Dong SL, Cui JJ. 2013. First transcriptome and digital gene expression analysis in Neuroptera with an emphasis on chemoreception genes in *Chrysopa pallens* (Rambur). *PLOS ONE* **8**: e67151. **DOI:** [10.1371/journal.pone.0067151](http://dx.doi.org/10.1371/journal.pone.0067151)

Li S, Zhu S, Jia Q, Yuan D, Ren C, Li K, Liu S, Cui Y, Zhao H, Cao Y, Fang G, Li D et al. 2018. The genomic and functional landscapes of developmental plasticity in the American cockroach. *Nature Communications* **9**: 1008. **DOI:** [10.1038/s41467-018-03281-1](http://dx.doi.org/10.1038/s41467-018-03281-1)

Lou F, Gao T, Cai S, Han Z. 2018. *De novo* assembly and annotation of the whole transcriptome of *Oratosquilla oratoria*. *Marine Genomics* **38**: 17-20. **DOI:** [10.1016/j.margen.2017.08.003](http://dx.doi.org/10.1016/j.margen.2017.08.003)

Mathers TC, Chen Y, Kaithakottil G, Legeai F, Mugford ST, Baa-Puyoulet P, Bretaudeau A, Clavijo B, Colella S, Collin O, Dalmay T, Derrien T et al. 2017. Rapid transcriptional plasticity of duplicated gene clusters enables a clonally reproducing aphid to colonise diverse plant species. *Genome Biology* **18**: 27. **DOI:** [10.1186/s13059-016-1145-3](http://dx.doi.org/10.1186/s13059-016-1145-3)

Miller JR, Koren S, Dilley KA, Harkins DM, Stockwell TB, Shabman RS, Sutton GG. 2018. A draft genome sequence for the *Ixodes scapularis* cell line, ISE6. *F1000Research* **7**: 297. **DOI:** [10.12688/f1000research.13635.1](http://dx.doi.org/10.12688/f1000research.13635.1)

Niehuis O, Hartig G, Grath S, Pohl H, Lehmann J, Tafer H, Donath A, Krauss V, Eisenhardt C, Hertel J, Petersen M, Mayer C et al. 2012. Genomic and morphological evidence converge to resolve the enigma of Strepsiptera. *Current Biology* **22**: 1309-1313. **DOI:** [10.1016/j.cub.2012.05.018](http://dx.doi.org/10.1016/j.cub.2012.05.018)

Oxley PR, Ji L, Fetter-Pruneda I, McKenzie SK, Li C, Hu H, Zhang G, Kronauer DJ. 2014. The genome of the clonal raider ant *Cerapachys biroi*. *Current Biology* **24**: 451-458. **DOI:** [10.1016/j.cub.2014.01.018](http://dx.doi.org/10.1016/j.cub.2014.01.018)

Papanicolaou A, Schetelig MF, Arensburger P, Atkinson PW, Benoit JB, Bourtzis K, Castañera P, Cavanaugh JP, Chao H, Childers C, Curril I, Dinh H et al. 2016. The whole genome sequence of the Mediterranean fruit fly, *Ceratitis capitata* (Wiedemann), reveals insights into the biology and adaptive evolution of a highly invasive pest species. *Genome Biology* **17**: 192. **DOI:** [10.1186/s13059-016-1049-2](http://dx.doi.org/10.1186/s13059-016-1049-2)

Poynton HC, Hasenbein S, Benoit JB, Sepulveda MS, Poelchau MF, Hughes DST, Murali SC, Chen S, Glastad KM, Goodisman MAD, Werren JH, Vineis JH et al. 2018. The toxicogenome of *Hyalella azteca*: a model for sediment ecotoxicology and evolutionary toxicology. *Environmental Science & Technology* **52**: 6009-6022. **DOI:** [10.1021/acs.est.8b00837](http://dx.doi.org/10.1021/acs.est.8b00837)

Rehan SM, Glastad KM, Lawson SP, Hunt BG. 2016. The genome and methylome of a subsocial small carpenter bee, *Ceratina calcarata*. *Genome Biology and* Evolution **8**: 1401-1410. **DOI:** [10.1093/gbe/evw079](http://dx.doi.org/10.1093/gbe/evw079)

Rider SD, Morgan MS, Arlian LG. 2015. Draft genome of the scabies mite. *Parasites & Vectors* **8**: 585. **DOI:** [10.1186/s13071-015-1198-2](http://dx.doi.org/10.1186/s13071-015-1198-2)

Rosenfeld JA, Reeves D, Brugler MR, Narechania A, Simon S, Durrett R, Foox J, Shianna K, Schatz MC, Gandara J, Afshinnekoo E, Lam ET et al. 2016. Genome assembly and geospatial phylogenomics of the bed bug *Cimex lectularius*. *Nature Communication* **7**: 10164. **DOI:** [10.1038/ncomms10164](http://dx.doi.org/10.1038/ncomms10164)

Sanggaard KW, Bechsgaard JS, Fang X, Duan J, Dyrlund TF, Gupta V, Jiang X, Cheng L, Fan D, Feng Y, Han L, Huang Z et al. 2014. Spider genomes provide insight into composition and evolution of venom and silk. *Nature Communication* **5**: 3765. **DOI:** [10.1038/ncomms4765](http://dx.doi.org/10.1038/ncomms4765)

Sasaki M, Akiyama-Oda Y, Oda H. 2017. Evolutionary origin of type IV classical cadherins in arthropods. *BMC Evolutionary Biology* **17**: 142. **DOI:** [10.1186/s12862-017-0991-2](http://dx.doi.org/10.1186/s12862-017-0991-2)

Shen J, Cong Q, Kinch LN, Borek D, Otwinowski Z, Grishin NV. 2016. Complete genome of *Pieris rapae*, a resilient alien, a cabbage pest, and a source of anti-cancer proteins. *F1000Research* **5**: 2631. **DOI:** [10.12688/f1000research.9765.1](http://dx.doi.org/10.12688/f1000research.9765.1)

Sikkink KL, Kobiela ME, Snell-Rood EC. 2017. Genomic adaptation to agricultural environments: cabbage white butterflies (*Pieris rapae*) as a case study. *BMC Genomics* **18**: 412. **DOI:** [10.1186/s12864-017-3787-2](http://dx.doi.org/10.1186/s12864-017-3787-2)

von Reumont BM, Campbell LI, Richter S, Hering L, Sykes D, Hetmank J, Jenner RA, Bleidorn C. 2014. A polychaete’s powerful punch: venom gland transcriptomics of *Glycera* reveals a complex cocktail of toxin homologs. *Genome Biology and Evolution***6**: 2406-2423. **DOI:** [10.1093/gbe/evu190](http://dx.doi.org/10.1093/gbe/evu190)

Werren JH, Richards S, Desjardins CA, Niehuis O, Gadau J, Colbourne JK, Werren JH, Richards S, Desjardins CA, Niehuis O, Gadau J, Colbourne JK et al. 2010. Functional and evolutionary insights from the genomes of three parasitoid *Nasonia* species. *Science* **327**: 343-348. **DOI:** [10.1126/science.1178028](http://dx.doi.org/10.1126/science.1178028)

Wu C, Twort VG, Crowhurst RN, Newcomb RD, Buckley TR. 2017b. Assembling large genomes: analysis of the stick insect (*Clitarchus hookeri*) genome reveals a high repeat content and sex-biased genes associated with reproduction. *BMC Genomics* **18**: 884. **DOI:** [10.1186/s12864-017-4245-x](http://dx.doi.org/10.1186/s12864-017-4245-x)

Zhao C, Escalante LN, Chen H, Benatti TR, Qu J, Chellapilla S, Waterhouse RM, Wheeler D, Andersson MN, Bao R, Batterton M, Behura SK et al. 2015. A massive expansion of effector genes underlies gall-formation in the wheat pest *Mayetiola destructor*. *Current Biology* **25**: 613-620. **DOI:** [10.1016/j.cub.2014.12.057](http://dx.doi.org/10.1016/j.cub.2014.12.057)
